# Flexible Membranes for Batteries and Supercapacitor Applications

**DOI:** 10.3390/membranes12060583

**Published:** 2022-05-31

**Authors:** Olga Glukhova

**Affiliations:** 1 Department of Physics, Saratov State University, 410012 Saratov, Russia; glukhovaoe@sgu.ru; 2 Institute for Bionic Technologies and Engineering, I.M. Sechenov First Moscow State Medical University (Sechenov University), 119991 Moscow, Russia

Modern portable electronic devices, roll-up displays and wearable systems for personal multimedia devices require flexible energy storage devices. Currently, the main sources of energy for portable electronics are lithium-ion batteries for high energy densities or supercapacitors for high power densities. To improve battery safety, the research and development of a fully flexible solid polymer electrolyte membranes is needed. These membranes must have good ionic conductivity to ensure efficient battery performance. The research and development of less-studied but effective ion exchange membranes for supercapacitors (e.g., proton exchange membranes (PEM) and anionic hydroxide exchange membranes (AEM)) also require improved scalable synthesis techniques, manufacturing techniques and an understanding of fundamental physical processes. The development of high-performance flexible batteries and supercapacitors relies heavily on innovative materials that have good electrical and mechanical properties.

The Special Issue “Flexible Membranes for Batteries and Supercapacitors” is devoted to fundamental research on the ion transfer process of flexible membranes and the problems of synthesis, development, and technologies for the design of membranes for electrochemical energy sources. There are six research articles in this Special Issue.

Li et al. [1] developed a noninvasive crop water sensor on the base of flexible graphene oxide. The sensor developed high sensitivity: under the measured voltage of 0.2 V and frequency of 100 Hz, the resistance changed between 6.7 × 10^5^ and 9.2 × 10^3^ with respect to the RH range of 11 to 95%. Under different humidity conditions, the sensor had different types of conductivity. The humidity monitoring sensitivity of the sensor reached 7945 Ω/% RH, and the response time was 20.3 s. The application of such a sensor in individual plant biology is obvious since such sensors allow the dynamical monitoring of water transpiration inside the plants. This approach can be an important tool for assessing the adaptability of crops to water stress and adjusting the reasonable conditions for the growth of crops. The authors estimated plant morphology before and after irrigation as well as the correlation between light-regulated transpiration and photosynthesis. 

Wang et al. [2] synthesized a novel attapulgite–sulfur–polypyrrole composite that was further applied as cathode material for Li–S batteries. The composite was examined with infrared spectroscopy, thermogravimetric analysis and scanning electron microscopy; then, electrochemical measurements were performed. The new material demonstrated 1175 mAh/g at a 0.1 C and retained a 44% capacity after 100 cycles. The role of attapulgite during the charge/discharge cycles was clearly confirmed. The pores and channels of attapulgite can be filled by polysulfides that inhibit the so-called “shuttle effect”. This finding makes the new material a very valuable block for Li–S batteries.

Ahmed et al. [3] reported a novel one-step approach for the preparation of STiO_2_ with a following fabrication of chitosan/STiO_2_ nanocomposite membranes for fuel cell application. The presence of SO_3_H forms proton-conductive pathways in the composite improves proton conductivity to 0.035 S·cm^−1^ when the STiO_2_ concentration reaches to 7.0 wt%. This value is close to the commercial Nafion 117 membrane (0.033 S·cm^−1^). The growth of STiO_2_ content also increases the tensile strength, which can reach 25.30 MPa. The enhanced thermal and mechanical stability, along with the significant proton conductivity, makes the chitosan/STiO_2_ nanocomposite a promising material for applications in proton exchange membrane fuel cells.

Cai et al. [4] fabricated a composite polymer anion exchange membrane with a sandwich structure. Each component played a crucial role for the membrane’s parameters. The embedded regenerated degreasing cotton improved the mechanical properties (tensile strength of 0.455 MPa and elongation at break of 82.13%) and alkali resistance (8 M KOH), while the outer layer’s poly-acrylic potassium served as the conducting phase for ion transmission. Furthermore, the considered composite demonstrated an electrochemical stability window of 2.0 V via the cyclic voltammetry curve test. These facts determine the successful application of the composite in Zn–Air batteries.

Mathematical modeling is also an important tool for the study of electrons and the transport properties of flexible membranes that can find applications in batteries and supercapacitors. Such calculations can predict the behavior of different materials and save the experimenters’ time. Works [5,6] by Glukhova et al. represent the atomistic simulation of modified carbon nanocomposites by the self-consistent-charge density-functional tight-binding (SCC-DFTB) method that determines the accuracy of ab initio methods but can be applied for supercells with much more atoms. 

The authors of [5] calculated the electrically conductive and electrochemical properties of hybrid films formed by bilayer graphene and single-walled nanotubes under axial stretching. Several supercells were considered that differed by the width of graphene layers and its overlap area. In the topological model with the narrowest graphene layers (two hexagons), the transformation of the planar bilayer graphene into a corrugated structure was observed after 10% elongation. Such transitions lead to a significant increase in electric resistance in the zigzag direction from 7.53 to 218 kΩ and a decrease in the quantum capacitance from 1000 to 200 F/g at 0 V. Conversely, for the topological model with the widest graphene layers (four hexagons), stretching decreases the difference between resistances in the zigzag and armchair directions and retains the dependence of the quantum capacitance on the applied voltage. The obtained results create a good background for the potential application of such composites in supercapacitors and batteries after experimental confirmation.

The combination of Fe_3_O_4_’s high theoretical capacity with the unique mechanic and conductive properties of graphene makes the graphene/Fe_3_O_4_ nanocomposite a high-demand material in the sphere of chemical power sources. Supercells with different mass ratios of components were considered in [6]. It was found that the formation of a composite with a mass ratio of m(Fe_3_O_4_):m(G) = 3:7 needs to overcome the lowest energy barrier (0.07 eV), while the formation of a 1:9 composite is the highest (0.21 eV). Herewith, the most stable compound corresponds to a 1:4 composite, for which the binding energy is 2.65 eV. The increase in the iron particles concentration leads to increasing Fermi levels from −4.42 eV for a 1:9 ratio to −3.78 eV for a 1:1 ratio. Quantum capacitance is also sensible to magnetite’s concentration. The highest value of quantum capacitance at 0 V was fixed for 1:1 ration and equaled to 583.52 F/g, which is much higher than for pure graphene (36.46 F/g). In addition, it was shown that by varying the concentration of magnetite, these electrode materials can be used in both hybrid and symmetric supercapacitors.

In conclusion, both the theoretical and experiment contribution of this Special Issue focus on the application of membranes in the field of batteries and supercapacitors. The synthesized new materials along with the developed methods and provided prediction could become important steps in the future development of flexible storage devices that become essential parts of our lives. 
